# Supplementary material for: Are carbon emissions trading and green financial instruments synergistic? -Comprehensive quantitative research based on content analysis
Source: PLoS One. 2024 Mar 7;19(3):e0298601. doi: 10.1371/journal.pone.0298601 (PMC10919601; doi:10.1371/journal.pone.0298601)
Supplement: S1 Data — (ZIP) [file pone.0298601.s001.zip › Data/地级市Z_F工作报告.zip!/║╙─╧/╨┼╤⌠╩╨/└┤╫╘╣1⁄2╓┌║┼í╛┬φ┐╦╩2╛▌═°í┐_2017.docx]

各位代表：

现在，我代表市人民政府，向大会报告工作，请予审议，并请各位政协委员和其他列席的同志提出意见。

一、过去五年工作回顾

过去的五年，是我市经济社会发展极不平凡的五年，面对复杂的外部环境和繁重的发展任务，我们在以习近平同志为核心的党中央坚强领导下，认真贯彻省委、省政府和市委的决策部署，团结带领全市人民，抢抓机遇，应对挑战，真抓实干，砥砺前行，实现了经济社会持续较快发展，较好地完成了市四届人大确定的目标任务。

过去的五年，是我市综合实力跨越提升的五年。五年来，全市生产总值年均增长9.1%，2016年总量跨过2000亿元，是2011年的1.6倍；一般公共预算收入94.7亿元、支出404.9亿元，分别是2011年的2.1倍和1.8倍；固定资产投资突破2200亿元，是2011年的2.3倍，总量居全省第5位；社会消费品零售总额981.5亿元，较2011年增长91.7%，总量居全省第5位；金融机构存款余额2692.3亿元、贷款余额1442.7亿元，分别是2011年的2.1倍和2.3倍，总量分别居全省第4位和第6位；非公经济持续健康发展，增加值对GDP的贡献率在60%以上。

去年是本届政府的收官之年，面对经济下行持续加大的压力，始终把稳增长作为重中之重，全市经济社会发展呈现稳中有进态势。全市生产总值同比增长8.3%，高于全省0.2个百分点，一般公共预算收入增长9.8%，规模以上工业企业增加值增长8.8%，固定资产投资增长9.6%，社会消费品零售总额增长11.5%，城乡居民收入分别增长6.8%和8.2%，较好完成了年度主要目标，实现了“十三五”良好开局。

过去的五年，是我市转型发展逐步加快的五年。三次产业结构不断优化，由2011年的26.2:41.6:32.2转变为21.9：39.6：38.5。农业供给侧改革深入推进，粮食产量连续11年超百亿斤，连续3年荣获全国粮食生产先进单位；优质水稻、弱筋小麦、茶叶、油茶、花木、食用菌、中药材等特色农业产业发展迅猛；培育省级农业产业化集群17个、省级农业产业化重点龙头企业55家，数量均居全省首位；信阳毛尖位居“中国十大茶叶区域公用品牌”第二位，品牌价值达59.9亿元，信阳获得“中国毛尖之都”殊荣。工业转型升级不断加快，规模以上工业企业完成增加值610.2亿元、主营业务收入突破2600亿元，高成长性制造业完成增加值303.2亿元，高技术产业完成增加值57亿元；绿色食品、新型建材、电子信息、现代家居、现代物流等主导产业总规模突破1200亿元，产业集聚区对全市规模以上工业企业增加值增长的贡献率达到98.7%。现代服务业快速发展，体育、旅游、文化、健康等产业进一步融合，累计实现旅游综合收入625.6亿元，年均增长21.3%，新县、商城县、浉河区成为国家全域旅游示范区创建单位，鸡公山国家自然保护区被评为“中国慢生活休闲体验区”；新建各类电子商务创业孵化园区17个，电商物流企业达到311家；国家集中连片推进农产品流通试点市建设顺利完成，国家级农产品冷链流通标准化试点市成功获批；交通银行、民生银行、中原农险近20家金融机构进驻信阳，村镇银行实现县区全覆盖；羚锐制药、华英农业完成再融资21.6亿元，黄国粮业、雄狮装备等4家企业登陆新三板；2016年资本市场直接融资较2011年实现翻番。

过去的五年，是我市改革开放不断深化的五年。重点领域改革不断深化，“放管服”改革深入推进，取消和下放行政职权399项；商事制度改革全面实施，新增市场主体7.6万户；不动产统一登记率先实施；投融资体制改革取得突破，推广政府和社会资本合作模式，储备PPP项目72个，总投资466亿元；圆满完成税收征管体制改革试点任务。国家农村改革试验区建设扎实推进，为全国农村改革提供了有益探索。对外开放持续深化，信阳海关建成通关，信阳铁路口岸、淮滨水运口岸等纳入省“十三五”口岸发展规划，跨境电商综合试验区建设全面启动，信阳茶文化节知名度和影响力不断扩大。实际利用外资年均增长12.8%，外派劳务2.1万人次，26家国内外500强企业来信阳投资。积极实施创新驱动战略，新增国家级高新技术产业化基地、可持续发展试验区、产业技术创新战略联盟和知识产权优势企业各1家，新增高新技术企业21家；全面实施8个省级重大科技专项，省科技进步一等奖实现零突破；培育中国驰名商标9个、中国地理标志证明商标4个、国家地理标志保护产品3个。

过去的五年，是我市城乡面貌明显改观的五年。编制完成信阳市城市总体规划并获批，启动中心城区控规及专项规划编制，稳步推进城乡一体化示范区建设。中心城区建设不断加快，新区建设成效显著，城市道路、供排水、供电、环保等基础设施不断改善，载体功能日益增强，棚户区和城中村改造有序推进，中心城区建成区面积达94平方公里，常住人口达87万人；县城建成区面积达226平方公里。全市城镇化率达44.4%，较2011年提高8.2个百分点。农村人居环境有效改善，首部地方性法规《信阳市传统村落保护条例》正式实施，创建省级生态村129个，郝堂村被住建部授予中国人居环境范例奖，以新集村、西河村、里罗城村为代表的众多美丽乡村，让人们看得见山望得见水记得住乡愁。我市相继获得全国绿化模范城市、国家卫生城市、全国文明城市提名城市等荣誉称号，连续八年入选中国十佳宜居城市，连续六届荣获中国最具幸福感城市。

过去的五年，是我市发展支撑显著增强的五年。全社会固定资产投资累计完成8955.1亿元，年均增长16.7%。交通路网建设全面突破，境内石武高铁、息淮固高速等重大交通项目建成通车，明港机场项目建设基本完成，息县至邢集高速公路、淮滨至息县航运工程淮滨段开工建设，G107和G312线绕市区段改线工程、许昌至信阳高速公路信阳段、沿大别山高速公路明港至鸡公山段、安阳至罗山高速公路上蔡至罗山段等即将开工建设，京九高铁（阜阳经潢川至九江段）纳入中国铁路网中长期规划并积极开展前期工作，宁西高铁建设可行性研究纳入国家中原城市群发展规划。土地资源保障有力，批准建设用地13万亩，实施土地综合整治项目15个，落实补充耕地11万亩。水利建设累计完成投资142.7亿元，全市923座大中小型水库完成了除险加固，开展了27条中小河流治理，新中国成立以来我省单体投资最大的水利工程出山店水库获批并开工建设，已完成投资66.2亿元。电力建设完成投资89.1亿元，农村电网加快改造升级，500千伏春申输变电工程建成投运。生态建设成效明显，新县、商城县、光山县、罗山县、浉河区纳入国家重点生态功能区，新县、商城县分别荣获国家、省级生态县称号，全市森林覆盖率达36.1%，分别高出全国、全省14.1和12.3个百分点；城市饮用水源水质达标率100%；大气污染攻坚战成效显著，空气质量全省第一，为全省生态建设作出了贡献。

过去的五年，是我市民生福祉持续改善的五年。五年累计民生支出1254.9亿元，是上个五年的2.4倍，更多群众分享到改革发展成果。脱贫攻坚强力推进，坚持“转扶搬保救”五条路径，创新“特色产业+园区+贫困户”等八种产业扶贫模式，实施3236户、12176人易地扶贫搬迁，建立贫困群众社会保障兜底机制，累计实现61.9万贫困人口稳定脱贫。就业形势保持稳定，累计新增城镇就业50.5万人，新增农村劳动力转移就业39.7万人；支持创业带动就业，吸引4万多名外出务工人员返乡创办各类实体3.1万个，带动就业近32万人。社会保障体系日趋健全，城乡居民基本养老保险和大病保险实现全覆盖，城镇企业退休人员基本养老金逐年提高，社会救助制度不断完善，城乡低保、特困人员救助供养标准逐步提升，电子社保示范城市建设扎实推进；住房保障体系基本形成，累计竣工保障房2.7万套，完成农村危房改造12.5万户。教育事业迈上新台阶，公办幼儿园和普惠性民办幼儿园覆盖率达87%，罗山县、光山县、新县义务教育均衡发展通过国家评估认定，高中教育质量连续多年保持全省领先，空军招飞工作连续4年位居全国地市首位，信阳农林学院成功升本，信阳学院挂牌成立。文化体育设施不断完善，成功举办国家登山健身步道联赛等一系列有影响的全国性体育赛事，群众文化生活更加丰富。卫生健康事业持续发展，基本药物制度全面实施，县级公立医院改革基本完成。平安信阳建设取得新成效，社会治安防控能力不断增强，突发公共事件应急体系逐步建立，金融风险有效防控，信访和安全生产形势总体平稳，群众安全感、满意度进一步提升。

过去的五年，政府自身建设切实加强，党的群众路线教育实践活动、“三严三实”专题教育和“两学一做”学习教育扎实开展。自觉接受市人大的法律监督和工作监督，主动接受市政协的民主监督，办理人大代表建议783件、政协委员提案1365件。支持民主党派、工商联和无党派人士参政议政。深化政务公开，全面公布政府权责清单，强化行政监察、行政复议、行政执法监督和审计监督。严格履行全面从严治党的政治责任，认真落实中央八项规定和省市若干意见，驰而不息纠正“四风”，严肃查处违纪违法案件，切实抓好中央和省委巡视反馈问题整改，政府系统廉政建设取得新成效。

各位代表！过去的五年，精神文明建设持续加强，爱国卫生运动广泛开展，公民道德水平和城乡文明程度明显提升。持续深化“双拥”共建，海军“信阳舰”入列命名，涉军维权的“信阳模式”被最高人民法院总结推广，连续五届荣获“全国双拥模范城”称号。驻信解放军和武警、消防部队在支持地方建设、抗洪抢险、维护稳定等方面作出了重要贡献。工会、共青团、妇联和科协、文联、残联、社科联、红十字会等人民团体在经济社会发展中发挥了不可替代的作用，民族宗教、外事侨务、对台工作全面加强，统计、物价、地质、气象、人防、农机、档案、地方志、机关事务、防震减灾、无线电管理等工作取得了新的成绩。

各位代表！过去五年取得的成绩来之不易，这是落实党中央、国务院和省委、省政府决策部署的结果，是市委坚强领导的结果，是市人大、市政协监督支持的结果，是全市人民同心同德奋力拼搏的结果。在此，我代表市人民政府，向全市广大工人、农民、知识分子，向解放军指战员、武警官兵、政法干警，向中直、省直机关驻信单位，向各民主党派，向全市新闻工作者，向在我市创业的境内外投资者、建设者及社会各界人士，致以崇高的敬意和衷心的感谢！

过去五年积累的经验弥足珍贵：一是必须始终把发展作为第一要务。产业集聚区发展壮大的历程告诉我们，必须适应把握引领经济发展新常态，立足信阳实际，找准发展定位，深入谋划主导产业，实施重大产业项目，促进经济平稳较快增长；二是必须始终把改革作为内生动力。国家农村改革试验区的实践探索启发我们，必须紧紧围绕重点领域和关键环节，着力破解束缚发展的体制机制障碍，集聚各类发展要素，合理配置各类资源，让改革的红利充分释放，让市场的活力竞相迸发；三是必须始终把生态作为最大优势。国家主体功能区建设试点示范市的成功创建昭示我们，必须坚持绿水青山就是金山银山的理念，不贪一时之利，不求一时之功，无论面临多大诱惑，始终做到经济发展服从生态保护，不断厚植生态环境新优势，开辟绿色发展新路径；四是必须始终把为民作为根本目的。打赢脱贫攻坚战的政治任务要求我们，必须坚持以人民为中心的发展思想，无论财力多么困难，始终把有限的资金用在增进民生福祉上，使发展更具公平性、普惠性，让人民群众有更多获得感、幸福感。

各位代表！在看到成绩的同时，也要清醒认识到我们面临的困难和问题，主要表现在：基础较差，发展困难大，结构性矛盾突出，农业大而不强，工业基础薄弱，第三产业发展还不够快；贫困人口基数大，精准扶贫精准脱贫任务非常艰巨；城乡发展不平衡，城镇化水平低，公共服务均等化水平不高，民生保障能力不强；少数部门和政府工作人员思想不够解放，创新能力不强，懒政怠政、推诿扯皮、执行不力等问题依然存在。对此，我们将采取有力措施，认真加以解决。

二、今后五年目标任务

今后五年，是信阳打赢脱贫攻坚战、全面建成小康社会的决胜期，也是信阳乘势而上、大有作为的重要战略机遇期。去年召开的市五次党代会指明了信阳未来发展的方向，开启了建设实力信阳、生态信阳、活力信阳、诚信信阳、清风信阳的新征程。当前，国家“一带一路”战略和河南省四大国家战略深入实施，特别是《大别山革命老区振兴发展规划》、中办国办《关于加大脱贫攻坚力度支持革命老区开发建设的指导意见》等政策相继落实，淮河生态经济带纳入国家“十三五”规划，为我市发挥优势、跨越赶超提供了难得机遇，全市上下思发展、谋发展、争发展的态势已经形成。同时，新一轮区域竞争日趋激烈，我市与全省先进地区的差距有逐步拉大的趋势，与我们处于同一发展梯次的黄淮四市后发赶超的势头十分强劲。因此，我们要时刻保持清醒头脑，进一步增强忧患意识，在新的五年拿出真劲头、硬措施，脚踏实地，大干苦干，才能缩小与先进地区的差距，才能在新一轮的竞争中不被淘汰，才能不负重托、交出一份无愧于全市人民的满意答卷！

按照市五次党代会作出的部署，今后五年政府工作的总体要求是：全面贯彻党的十八大和十八届三中、四中、五中、六中全会精神，深入学习贯彻习近平总书记系列重要讲话精神，认真落实党中央治国理政新理念新思想新战略，统筹推进“五位一体”总体布局，协调推进“四个全面”战略布局，始终坚持发展第一要务，牢固树立和贯彻落实创新、协调、绿色、开放、共享的发展理念，打好“四大攻坚战”，着力构建区域性中心城市、充满活力的生态经济先行区和全国有影响力的交通枢纽、信息枢纽、物流枢纽，努力建设“五个信阳”，全面建成小康社会，为全面实现现代化奠定坚实基础。

按照市五次党代会描绘的宏伟蓝图，今后五年政府工作的主要奋斗目标是：

——综合实力再上台阶。在提高发展平衡性、可持续性的基础上，到2020年全市生产总值和城乡居民人均收入比2010年翻一番以上，生产总值年均增速高于全省平均水平1个百分点以上，经济社会发展主要指标接近全省平均水平，综合实力进入全省第二方阵前列。

——脱贫攻坚决战决胜。贯彻落实中央“六个精准”“五个一批”精准扶贫精准脱贫要求，落实政策，完善机制，加大投入，在激发内生动力、改善发展条件、提升扶贫实效、促进均衡发展上狠下功夫，确保到2019年农村贫困人口全部实现稳定脱贫，贫困村、贫困县全部摘帽，退出贫困序列。

——改革开放全面深化。重要领域和关键环节改革取得决定性成果，基本形成系统完备、科学规范、运转有效的制度体系。交通、信息和物流枢纽影响力辐射全国，对外开放、招商引资活力进一步激发。创新创业质量明显提升，创新驱动成为引领发展的主要动力。

——城乡面貌焕然一新。围绕构建区域性中心城市，全面实施百城建设提质工程，建立科学的规划、建设、管理机制，推动交通一体、产业链接、服务共享、生态共建，不断提高辐射带动能力，市中心城区吸纳承载人口100万人以上，全市常住人口城镇化率达52%以上。

——生态环境日趋优美。大气和水环境质量继续位居全省前列，森林覆盖率达到38.5%以上，自然生态更加秀美，城乡环境更加宜居，所有县城达到省级园林城市标准。“一城三农五生态”空间开发格局初步形成，生态农业、生态工业、生态服务业加快发展，成为全省重要的生态经济先行区。

——群众生活持续改善。基本公共服务均等化程度进一步提升，广覆盖、保基本、多层次、可持续的社会保障体系基本建立，社会就业更加充分，低收入群体生活质量明显改善，基本养老保险参保率达95%以上，群众生活更有保障、更有尊严、更加幸福。

——社会更加和谐稳定。社会建设全面加强，精神文明、政治文明和民主法制建设取得明显成效，社会公平正义充分彰显。社会管理创新能力进一步提高，公共安全体系更加完备，法治建设、平安建设扎实推进，社会大局和谐稳定。

各位代表，实现上述目标，事关人民福祉，事关发展全局，必须凝神聚力，久久为功。我们坚信，有省委、省政府和市委的正确领导，有全市人民的共同努力，今后五年的发展目标一定能够实现！信阳的明天一定会更加美好！

三、2017年工作安排

今年是党的十九大召开之年，也是新一届政府的开局之年，做好今年的政府工作至关重要。

全市经济社会发展的主要预期目标是：生产总值增长8.5%，一般公共预算收入增长8%，全社会固定资产投资增长12%，社会消费品零售总额增长11%，进出口总值保持稳定，城镇新增就业9.6万人以上，常住人口城镇化率提高1.5个百分点以上，居民消费价格涨幅3%左右，城乡居民收入与地区生产总值同步增长，完成省下达的节能减排、环境保护等约束性指标任务，发展质量和效益明显提高，供给侧结构性改革取得实质进展。

围绕上述目标，市政府将重点抓好以下九个方面的工作：

（一）强力推进脱贫攻坚，持续补齐发展短板。把脱贫攻坚作为头等大事和第一民生工程，坚持以脱贫攻坚统揽经济社会发展全局，狠下“绣花”功夫，确保完成年度目标任务。

扎实整改。以督查巡查反馈问题的整改为契机，对脱贫攻坚工作进行一次全面“体检”，确保脱贫攻坚方向明、问题清、路子对。围绕漏识、错识、误退三个关键环节，抓好县乡村三级档案和贫困户明白卡规范管理。对查摆出来的问题深刻分析，找准病根，对症下药，同时注重举一反三，避免同样问题再发生。认真落实重点整改、全面整改和系统整改三大任务，严格落实“六个一”要求，把“精准”原则贯穿整改全过程，做到信息采集精准、建档立卡精准、实施帮扶精准、脱贫退出精准，确保实现“三个零差错”和“三个明显提高”的目标。

精准施策。做实产业扶贫，构建金融助推扶贫机制，把金融资金引向能带动群众稳定增收的致富产业和龙头企业中来，实施好“多彩田园”示范工程和“牵手”工程，建立市场主体与贫困户的利益联结机制。坚持“外转”“内转”相结合，引导有劳动能力的贫困群众通过就业实现脱贫。推进易地扶贫搬迁，按照“四靠”原则建设安置点，统筹考虑群众搬迁后的生产生活问题，确保搬得出、稳得住、能发展、可致富。开展生态综合补偿试点，统筹使用生态资金，将贫困人口就地转为生态保护人员。统筹整合财政涉农资金，发挥财政资金扶贫的最大效益。对建档立卡的贫困家庭学生免除普通高中学费、住宿费，确保其不因家庭困难而失学。建立困难群众大病补充医疗保险制度，实现医疗救助更大范围覆盖。进一步加强农村低保制度与扶贫开发政策的有效衔接，提高“鳏寡孤独痴残”六类兜底对象农村低保补助水平。同时，坚持扶贫与扶志相结合，激发贫困群众内生动力；高度重视“两非”人员脱贫，加大对非贫困县、非贫困村贫困人口扶持力度，促进脱贫攻坚工作均衡发展，在全面小康的道路上，决不让一个贫困村落伍，决不让一名贫困群众掉队！

压实责任。严格落实县级党委政府第一责任、乡村两级具体责任，市直单位、村级责任组、驻村工作队、第一书记精准帮扶责任。完善脱贫攻坚督查巡查机制，整合督查资源，实施常态化暗访检查。抓好县乡扶贫开发成效考核和实绩考评，强化考评结果运用。加强扶贫资金监督检查，严查贪污、截留、挪用行为。严格执行贫困退出标准和退出程序，确保脱贫成效，不折不扣兑现军令状。

（二）强力推进项目建设，持续筑牢发展支撑。扎实开展“项目建设年”活动，以项目建设引领全局，确保完成各类项目年度投资计划。

积极谋划。瞄准国家政策导向、投资趋向和产业方向，提高项目谋划水平，在完善城市功能、改善民生和产业发展等方面，储备一批重大项目，形成梯次结构，确保常态化储备1000亿元以上。扎实推进项目前期工作，争取更多项目纳入中央和省级项目库，达到可批、可建条件。做好京九高铁、宁西高铁、淮滨至内乡高速公路豫皖省界至淮滨段、潢川机场、河南省大别山革命老区引淮供水灌溉工程、张湾水库、袁湾水库等一批重大项目前期工作，争取早日开工建设。

加快建设。抓好187个省、市重点项目建设，落实“5818”扩大有效投资行动，完成投资335亿元以上。全力攻坚中心城区30个重点项目，加快推进火车站北广场等11个已开工项目建设进度，确保沿大别山高速公路明港至鸡公山段、G107和G312绕市区段改线工程等19个项目全部开工；今年11月底，许昌至信阳高速公路信阳段、安阳至罗山高速公路上蔡至罗山段也要开工。加快重大工程建设，确保明港机场等23个项目竣工投运；推动出山店水库、山信食品工业园等88个续建项目建设；力促河南五岳抽水蓄能电站、信阳淮滨港等103个项目开工。抓好明阳碲化镉太阳能光伏发电系统等121个产业转型升级项目建设，完成年度投资104.3亿元，全面提升中高端产品供给水平。持续抓好城乡污水垃圾处理、贫困村基础设施、公共卫生、义务教育扩容等民生和社会发展项目实施。

强化保障。创建新型城镇化发展基金、产业发展基金，持续开展还贷周转金业务，解决百城建设提质工程和实体企业融资难问题。依法依规做好征地拆迁工作，加大土地收储力度，盘活存量和低效闲置土地，保障重大项目用地需求。改进完善联审联批制度，推进行政审批流程优化再造，畅通项目审批“绿色通道”。探索政府购买项目评审服务，最大限度缩短审批时限。引进投融资平台建设、项目谈判等方面的咨询公司和专业人才，为项目引进、合作提供智力保障。

完善机制。实行专人专班，安排专项经费，做好项目前期工作。继续实施市级领导分包联系重大项目制度、周例会制度、现场办公会制度，协调解决项目建设难点问题。强化项目督查，对所有重点项目进行全程督办，通过新闻媒体跟踪报道，人大、政协及社会各方监督，倒逼责任落实。健全考评机制，以项目推进论实绩，以项目建设显才干。

（三）强力推进工业发展，持续转变发展方式。坚定不移走工业立市、工业强市之路，做大总量，提升质量，确保规模以上工业增加值增长9.5%以上，完善政策，大胆探索，再掀支持工业、发展工业新热潮。

提升集聚水平。围绕提高产业集聚度和投入产出率，推动产业集聚区提质增效，引导产业集聚区集约化、差异化发展，完成固定资产投资1000亿元以上，主营业务收入达到2200亿元，实现二星级产业集聚区零突破。支持潢川经济技术开发区申报国家级经开区，推动符合条件的产业集聚区晋升为省级开发区。推进服务业“两区”建设，确保市商务中心区等4家服务业“两区”晋升为省一星级，力争其他服务业“两区”达到省定门槛标准。

创新招商方式。围绕我市资源禀赋和产业基础，坚持引资、引技、引智并举，着力引进科技含量高、带动能力强的大企业、大集团，全年完成招商引资310亿元以上。继续实行二分之一工作法，大员上前线招商；设立招商联络处，持续开展驻地招商；组织专业队伍，开展集群招商、前瞻性招商、市场招商、以商招商、“资本+产业”招商；充分发挥商会和行业协会的作用，拓宽招商引资渠道；大力实施“回归工程”，将在外信阳籍企业家和成功人士作为招商重点，以亲情、乡情为纽带，引导其返乡创业。抢抓贫困地区企业上市的“绿色通道”政策机遇，争取更多企业落户信阳。

发展主导产业。实施产业集群提质行动，推动产业链向两端延伸，价值链向高端延伸，逐步打造百千亿级优势产业集群。加快培育绿色食品千亿级产业集群，新型建材、电子信息、纺织服装、装备制造、生物医药等百亿级产业集群。

扶植重点企业。重点扶植20家企业做大做强，坚持“一企一策”，在项目建设、技术创新、战略重组、上市融资、市场开拓等方面给予扶持。支持黄国粮业、春泉园林尽快上市，支持开源环保、雄狮装备、盛高集团、赛思医疗、白鲨针布做上市筹备，支持天一窗业、君子林服饰等企业在新三板挂牌。加快信阳钢铁年产40万吨钢渣微粉、舜宇光学月产4000万片旋涂滤光片、煜锋电子年产400万片液晶显示模组等项目建设。推进明阳风电新能源装备制造基地、信阳林志科技孵化园、毅辉智能制造产业园二期、华仪开关二期、禹王水工二期、谷麦光电科技园一期等项目建设。

强化协调服务。完善常态化的协调机制，加强工业运行监测、预警、协调。落实市级领导联系企业制度，建立企业和基层评议政府部门制度，发挥企业服务110热线作用，健全企业反映问题快速办理机制，帮助扶持企业发展、帮助推进项目建设，促进民生改善、促进经济增长。依法依规减免企业负担，集中解决一批招商引资企业的历史遗留问题。尊重企业家创造，培育企业家精神，着力构建“亲”“清”政商关系，营造重商、亲商、安商的社会氛围。

（四）强力推进农业改革，持续培育发展动能。以调整优化农业结构和深化农村配套改革为重点，以创建高效生态经济示范区为抓手，深入推进农业供给侧结构性改革，持续增加农民收入、保障有效供给。

调整农业结构。重点发展优质粮油、优质畜禽、优质水产及茶叶、油茶、花卉、苗木等优质特色产业，加快推进布局区域化、经营规模化、生产标准化、发展产业化。调优粮食品种。落实粮食安全责任制，确保粮食综合生产能力不降低；发展优质水稻、优质弱筋小麦各100万亩、无公害蔬菜40万亩；引导畜禽标准化养殖和水产生态养殖，培育省级以上规模畜禽养殖示范场3个、名特水产养殖面积达到10万亩。调响特色品牌。叫响“信阳菜”“毛尖茶”“信阳米”“弱筋麦”“黄国粉”“山茶油”“南湾鱼”“华英鸭”“詹氏蜜”“万富油”等信阳知名地域农产品品牌，推进罗山县国家农产品质量安全县试点工作，支持息县“中国生态主食厨房”建设。调大经营规模。壮大各类新型农业经营主体，力争新发展示范家庭农场和农民专业合作社各40家、市级农业产业化重点龙头企业20家，培育新型职业农民1万人以上。调长产业链条。推进农产品初加工、精深加工及农副产品综合利用，规划建设现代农业产业园9个；推进优质稻米、弱筋面制品、植物油及水产品、林产品等农业产业化集群发展和产业链延伸，新增农业产业化集群7个；因地制宜发展立体种养、观光旅游、休闲体验农业等多功能化农业产业。

深化“三农”改革。推进国家农村改革试验区建设，启动重要农产品收入保险试点。落实各项惠农补贴政策，健全公益林补偿标准调整机制。深化农村集体产权制度改革，完成农村土地确权登记颁证，稳妥推进土地集体所有权、农户承包权、土地经营权“三权分置”配套改革，探索“资源变资产、资金变股金、农民变股东”模式，增加农民财产性收入。完成集体林权制度改革和9个国有林场改革任务。推进小型水利工程管理体制改革。深化供销改革，筹建信阳供销集团。创新农村金融，筹建信阳市农业信贷担保公司，在新县、光山县、商城县、淮滨县开展“农贷宝”试验，为农村提供便捷高效的金融服务。

筑牢农业基础。加强农田水利基本建设，持续开展“信阳精神杯”竞赛，新增改善灌溉和除涝面积167万亩，治理水土流失面积160平方公里。完成淮干一般堤防加固工程64公里，开工建设南湾、鲇鱼山、泼河、石山口4处大型灌区工程，新建高标准粮田60万亩以上。实施主要农作物全程机械化推进行动，争取主要粮食作物耕、种、收综合机械化水平达到75%以上。继续实施农村安全饮水巩固提升工程，今年再解决34.7万农村人口饮水安全问题。完成316个中心村电网改造升级，实现574个贫困村通动力电。新建农村公路1472公里、桥梁1659延米。加快农村宽带网络、4G通信网络建设。做好第三次全国农业普查。

（五）强力推进三产繁荣，持续提升发展水平。以满足需求为导向，通过消费转型升级，推动现代服务业向绿色化、特色化、融合化方向发展，打造经济增长新引擎。

壮大旅游产业。围绕“红”“绿”两大优势资源，全面落实《信阳市“十三五”旅游产业发展规划》。完成鸡公山、灵山和黄柏山创建国家5A级景区申报，支持南湾湖风景区创建国家生态旅游示范区，加快新县、商城县、浉河区创建国家全域旅游示范区建设。突出信阳的茶、湖特色，着力打造环南湾湖茶乡风情游精品线路，建设茶山标准化旅游示范点20个。实施“品信阳菜、喝毛尖茶”工程，推广“名菜、名吃、名厨、名店”，促进“信阳菜”标准化、品牌化、产业化建设；发展生态茶园、有机茶园，推广机械化、标准化生产，促进茶旅产业融合。全力推进新县全域旅游扶贫开发暨特色小镇、平桥区城阳城国家遗址公园、鸡公山“一园十三景”、光山县大苏山国家森林公园等项目建设。大力发展红色旅游，加强新县鄂豫皖苏区首府、罗山县何家冲红二十五军长征出发地、光山县王大湾会议会址、浉河区四望山革命遗址等红色文化资源保护开发，着力构建全国知名的红色旅游目的地。加快发展城市旅游，构建以浉河新八景为基础、以百花园区域为文化中心的城市旅游带。完成南湾湖、出山店水库、北湖风景区区域旅游规划和出山店至市区旅游带规划编制工作。实施乡村旅游倍增计划，在全市每个乡镇创建1-2个美丽乡村示范村。力争实现旅游接待人数3200万人次，旅游综合收入突破200亿元。

发展康养产业。重点谋划和打造生命健康服务业、生命健康制造业和健康农业三大板块，构建“医、药、养、健、游”五位一体产业链，谋划建设国家生命健康产业创新示范区，争创国家级康养产业试点市。抓好羊山体育中心“一校一馆”及中心城区健身场地设施建设，打造城市15分钟健身圈。引进社会力量建设一批规模化、连锁化养老机构，推动老年人日间照料中心等社区养老服务机构建设。完成中心城区集中供热一期工程，着力培育以远大住工为代表的装配式绿色建筑产业基地，加快推进和兴阳光城和义乌商贸城等城市综合体建设，同时提高商贸、餐饮、住宿、家政、物业等生活性服务业水平，不断提升康养产业的综合配套能力。

做强物流产业。发挥我市交通枢纽优势，依托铁路、公路网络，发展陆路物流；以明港机场为核心，谋划建设信阳空港经济区，发展航空物流；以淮滨港为核心，加快建设淮滨临港经济区，发展水运物流。扎实开展国家级农产品冷链流通标准化试点工作，推进特色物流发展。在金牛物流产业集聚区实施智能化改造提升工程，打造国家级示范物流园区。以市中心城区和潢川县为核心，逐步形成“双核”驱动的多式联运物流大格局。

提升信息产业。加快城乡宽带网络建设升级，实现城乡全覆盖。推动电子信息业、软件服务业等产业发展，加快建设北斗信息产业园、榕基软件园。大力实施阿里巴巴“农村淘宝”和京东农村电商项目，畅通网货下乡和农产品进城双向通道；持续实施电子商务示范基地和示范企业创建工程，创建省级电子商务示范企业3家以上。推进“互联网+”服务业、制造业融合创新，建设“互联网+”政务公共服务体系，加快智慧城市建设，进一步提高教育、交通、医疗、养老等民生领域信息化服务水平。

（六）强力推进城乡统筹，持续拓展发展空间。推进以人为核心的新型城镇化，加强城镇规划建设管理，展现城市特色，提升城市品位，打造美丽乡村。

强化规划引领。完成城市规划区控规编制，做好城市商业中心区、羊山新区、信阳高新区、南湾和浉河两岸等重要片区城市设计。开展中心城区城市综合交通、水系、绿地系统等19个专项规划编制。严格执行规划，坚决依法治理违规建筑，切实维护规划的严肃性。精细化管理用地指标，高度垄断中心城区土地一级市场，严格保护山水格局，合理控制中心城区开发强度。

提高建设标准。开展城市“双修”，实施新二十四大街、信阳火车站北站房和北广场区域改扩建、新十八大街贯通等工程，提升中心城区与外围交通环线衔接能力；实施新七大道延长段、新五大道西延下穿铁路桥、浉河2号桥、浉河步行桥等一批路、桥工程，提升新老城区的畅通能力；加快推进新十六大街前进广场、申碑路、浉河公园东门等一批城市停车场建设。大力发展城市公共交通，做好公交场站建设和公交车更新配备。推进海绵城市建设，加快建设羊山森林植物园、羊山公园、两河口湿地公园等一批公园绿地。支持潢川撤县建市，建设副中心城市。以光山县、淮滨县为示范，精心组织实施各县百城建设提质工程。推进平桥区明港镇、光山县马畈镇等扩权强镇试点建设，做好特色小镇申报工作。全面开展农村“双治”，持续改善农村人居环境。加强传统村落保护，传承历史文化，加快新县丁李湾、光山县董湾等建设国家民俗民居示范村。

提升管理水平。完善城市配套功能，着重推进硬件设施建设。加强城市市容管理，坚持城区全天候、全覆盖清扫保洁；深入开展“畅行信阳”交通改善行动；强力开展垃圾乱倾乱倒、摊点乱摆乱设、车辆乱停乱放、建筑乱搭乱建等整治；统筹抓好城市干道、商业街区、住宅小区、城乡接合部、城市视野区等重点部位绿化、净化、亮化，确保全国卫生城市复核和国家园林城市复检顺利通过，全国文明城市创建一举成功。强化城市历史文化保护，延续城市文脉，保留城市记忆。积极践行社会主义核心价值观，培育城市精神，提升市民素质。有序推进农业转移人口市民化。

（七）强力推进环境治理，持续厚植发展优势。坚持保护与治理相结合，加大治气治水力度，让信阳蓝天常驻、碧水常流、青山常在。

深化专项治理。持续打好大气污染攻坚战，坚决打好水污染防治攻坚第一战。继续严格落实“六控”措施，突出抓好秸秆禁烧和禁放区烟花爆竹禁燃禁放，确保完成“三治本、三治标”目标任务，确保PM10年均浓度在84微克/立方米以下，PM2.5年均浓度在58微克/立方米以下，优良天数达到215天以上，圆满通过终期空气质量考核。扎实推进水污染防治，确保11个地表水断面水质达标，城市集中式饮用水源地水质优于Ⅲ类比例总体达到98%，地下水质量保持稳定，消除城市建成区内黑臭水体。对流入浉河的15条城市内河进行治理，关闭南湾湖和浉河中心城区段汇水区内畜禽养殖场。加强沿淮流域环境治理，全面完成淮河干流、潢河、浉河、白露河流域年度防治任务，实施好市第二污水处理厂等20个年度重点工程。

加快生态建设。推进国家级森林城市创建和国家储备林基地建设，大力实施退耕还林、天然林保护、生态修复等重大生态工程，完成植树造林和森林抚育75.7万亩，在5个国家重点生态功能区造林24.3万亩。在南部山区和中部丘岗区，建设水源涵养林和水土保持林体系；在北部平原生态涵养区，完善和提升农田防护林体系；在沿淮生态保育带，大力推进淮河水源涵养林、淮河生态防护林和干流防护林带建设。积极推进生态原产地产品保护及其示范区建设。

完善长效机制。全面落实?“河长制”，保护治理好河湖水系。毫不动摇执行产业发展和区域开发环境保护负面清单，全面强化环境准入制度，完善污染企业退出机制。加快网格化环境监管体系、环境监测能力和环境执法能力建设，提升常态化监管水平。实行最严格的源头保护、损害赔偿和责任追究制度，建立健全生态环境保护激励机制，探索用能权、用水权、排污权、碳排放权初始分配和有偿使用及交易制度。

（八）强力推进改革开放，持续激发发展活力。坚持改革驱动、开放带动，稳步落实各项改革任务，加强开放合作，进一步激发经济发展的动力和活力。

推进投融资体制改革。完善政府投资管理配套办法，健全非经营性政府投资项目代建机制，推进政府投资项目分类管理，全方位、全过程规范政府投资行为。鼓励社会资本参与公共领域项目建设，大力推广PPP模式，积极推进符合条件的PPP项目通过资产证券化方式实现市场化融资，提高资金使用效率。今年，争取羊山公园等20个项目进入国家和省PPP项目库。推动政府投融资平台去行政化改革，健全法人治理结构，发挥发投公司和华信投资公司的平台作用，加快城投、水投公司实质性运作步伐，筹建交通、文化旅游等专业投融资平台，创新以市带县模式，整合行业、领域资源，依法规范运作，增强融资能力。

深化重点领域和关键环节改革。进一步简政放权，再精简一批行政审批、中介服务审批事项。深化商事制度改革，推进“多证合一”。深化国有企业改革，推动企业资产重组，加快剥离国有企业办社会职能。完成城管执法体制改革，推进执法重心下移和执法事项属地化管理。继续做好国税、地税征管体制综合改革，完善综合治税管理体系。进一步完善预算管理和非税收入管理等方面的改革，持续优化支出结构。大力发展普惠金融，深化金融产品和服务模式创新。全面完成农村信用社改制组建农商行工作。统筹推进环保、价格、教育、科技、文化等领域改革。

扩大对外开放。积极推进信阳保税物流中心（B型）、跨境电商综合试验区等项目建设，推动开放平台功能融合，协调联动。加快骏畅智能科技年产300万部手机项目建设，支持卢氏茶业继续开拓海外市场，推动企业扩大出口。主动融入“一带一路”战略，重点培育以茶叶、电子产业和羽绒为主的出口基地，开拓“一带一路”沿线市场。建立国际贸易“单一窗口”管理制度，推进通关“一体化”，提高对外开放综合服务能力。全年实际利用外资增长6.9%以上。

提升创新创业能力。抓好创新平台建设，发挥高等院校、科研院所对企业技术创新的源头支持作用，加快建设一批国家级、省级工程技术研究中心和重点实验室；推进产学研合作，支持大中型企业建立研发机构，力争新增各类创新平台5家以上；支持淮滨县麦德隆公司申报河南省弱筋小麦产业技术创新战略联盟，加快建设国家粘土岩石类非金属矿产品质量监督检验中心。积极搭建创业载体，推进创业孵化园区和新型众创空间建设，筹建信阳大学科技园，支持信阳高新区与信阳师院联合申报国家级众创空间。实施“信阳英才”计划，大力培育引进创新创业人才、优秀企业家、领军人物及团队；鼓励支持返乡农民工和大学生等群体，返乡下乡创业，培育创业示范县、示范乡镇、示范园区和示范项目。调整创业担保贷款实施办法，充实创业贷款担保基金，扩大财政贴息范围，支持大众创业万众创新。

（九）强力推进民生改善，持续落实发展共享。优先保障和改善民生，坚持把财力向民生领域倾斜，打牢共享发展基础。

办好十件实事。坚持从群众的实际需求出发，积极回应广大群众的期待，今年集中精力办好以下十件实事：第一件：新建、改扩建6所中小学校及幼儿园，缓解中心城区大班额和入学难问题；第二件：打通平桥区富民路、平三路、平东南路和浉河区兵站路等城市断头路，缓解中心城区交通拥堵；第三件：完成中心城区所有背街小巷的整修，达到“路平、灯明、墙净、植绿”基本要求；第四件：新建公厕及垃圾中转站30座，升级改造公厕及垃圾中转站30座，改善中心城区卫生和居住环境；第五件：对浉河中心城区地段排污口实施截污，解决污水直排浉河问题；第六件：完成9个农贸市场的新建和升级改造，推进中心城区标准化农贸市场建设；第七件：完成市、县156所学校食堂升级改造并安装网络视频监控，实现即时监督、远程监控和零距离监管；第八件：对全市农业灌溉基本水费实施财政补贴，免除农民自缴基本水费；第九件：提高全市城乡居民和城镇职工医保标准，城乡居民医保和新农合最高支付限额统一提高到55万元，城镇职工医保最高支付限额提高到26万元；第十件：全市完成天然气替代煤、电替代煤改造10万户以上，提高清洁能源使用率。这十件实事是市政府对全市人民的庄严承诺，不管有多大困难，必须保质保量完成；十件实事可督导、可考核、可追责，已向全市人民公开，接受全市人民监督。

做好就业托底。严格落实就业再就业政策，强化技能培训，解决好农村富余劳动力、城镇失业人员就业再就业问题，保障零就业家庭至少一人实现就业，城镇新增就业9.6万人以上，新增农村劳动力转移就业4.6万人以上。

完善社保体系。完成社会保障“一卡通”发放任务，推进“互联网+人社”电子社保示范城市建设。继续提高退休人员基本养老金，确保按时足额发放。全面完成全民参保登记，按时完成机关事业单位养老保险制度改革和城乡居民医保整合。稳步提高低保和特困人员救助供养标准。加大保障性安居工程建设力度，持续开展中心城区和县城棚户区改造，完成省定目标任务。

发展社会事业。坚持教育优先发展战略，继续实施第三期学前教育行动计划和“学生饮用奶计划”；加快推进“全面改薄”、义务教育扩容、寄宿制学校建设，统筹推进义务教育均衡发展；加快信阳大别山高中建设和信阳高中、市第三实验高中改扩建，尽快普及高中阶段教育；深入实施职业教育攻坚计划二期工程；支持大别山艺术高等专科学校和信阳航空职业学院申办建设；支持信阳师范学院等高等院校创建区域性优势大学或学科；全面实施乡村教师支持计划。发展文化事业，健全四级公共文化设施，广泛开展“百城万场”“中原文化大舞台”等文化惠民活动；加强文物和非物质文化遗产保护利用，深入开展传统文化进校园活动；推进全民阅读，建设书香信阳。启动城市公立医院改革，深化县级公立医院改革，加快建立分级诊疗制度；提升中医服务能力，推进中医堂、中医馆建设；加快市中心医院羊山分院、市儿童医院、市中医院高新区分院、市传染病医院项目建设，完成圣德医院建设；加强生育全程妇幼计生健康服务，积极落实全面两孩政策。举办好第五届全民运动会。

创新社会治理。加强平安信阳建设，加快网格化管理和服务向基层延伸，完成治安视频监控系统建设年度任务，构建立体化社会治安防控体系。严格落实安全生产责任制，防范重大安全生产风险，坚决杜绝重特大安全事故发生。落实“四个最严”要求，严防严管严控食品药品安全风险。加强应急救援及防灾减灾工作。整合公共资源交易平台，启动电子招标系统。推进社会信用体系建设。落实信访工作责任制，加强社会矛盾排查化解，妥善处置退役士兵安置遗留问题，及时解决群众合理合法诉求，确保社会和谐稳定。

加强国防教育和国防后备力量建设，支持军队改革，支持驻信解放军、武警部队、民兵和人民防空建设，深入开展“双拥”共建，推进军民融合深度发展，巩固军政军民团结。支持工会、共青团、妇联和科协、文联、残联、社科联、工商联、红十字会等人民团体发挥桥梁纽带作用，全面落实民族宗教政策，切实做好外事侨务、对台工作。继续抓好统计、物价、邮政、通信、地质、气象、档案、地方志、防震减灾、机关事务、市场发展、无线电管理等工作。

四、强化政府自身建设

加强和改进政府工作，勇于解放思想，重在实干为民，推动作风建设常态长效，树立为民务实清廉的政府形象。

（一）强化“四个意识”。牢固树立“四个意识”特别是核心意识、看齐意识，始终同以习近平同志为核心的党中央保持高度一致。严守政治纪律和政治规矩，保持政治定力，持续巩固提升“两学一做”学习教育成果，认真贯彻党的基本路线和方针政策，不折不扣贯彻落实好党中央、国务院和省委、省政府及市委的决策部署。

（二）勇于解放思想。坚持把解放思想作为应对新挑战、开创新局面的法宝，自觉用新的理念指导和促进工作。重视和加强学习，破除单凭经验做事、固步自封的旧观念，不断用新的理论武装头脑；自觉同先进地区对标，高树目标，自我加压，把影响发展的思想根源找准，把制约发展的条条框框打破。探索建立长效机制，真正把解放思想贯穿到政府工作的各个环节，体现到经济社会发展的方方面面。今年，重点要在产业发展、项目建设、投融资体制改革等方面有所突破。

（三）突出实干为民。坚持民生为先，破解难题，做到民有所呼，我有所应。持续强化作风建设，履职尽责，干字当头。对确定的目标，说了就算，定了就干，干就干好，干就干成。注重务实高效，提升工作效率，提高工作标准。完善机制，落实责任，进一步改进绩效考核办法，强化失职约谈、问责、追责，严肃查处懒政怠政和为官不为；认真落实“三个区分开来”，为敢于担当的干部担当，替敢于负责的干部负责，真正形成责任倒逼和正向激励双重机制，以钉钉子精神确保各项工作落到实处。

（四）严格依法行政。牢固树立法治意识，把依法行政、依法办事原则贯穿到政府工作全过程，严格按照法定权限和程序行使权力、履行职责。加强政府工作人员法制培训，提高法治素养。坚持和完善公众参与、专家论证、风险评估、合法性审查和集体决策相结合的决策程序。全面落实行政执法责任制，严格、规范、公正、文明执法。自觉接受市人大法律监督、市政协民主监督和新闻媒体、社会公众监督，认真办理人大代表建议和政协委员提案，让行政权力始终在法治轨道和阳光下运行。

（五）坚持廉洁从政。落实全面从严治党主体责任和“一岗双责”。强化行政监察和审计监督，实现公共资金、国有资产、国有资源和领导干部履行经济责任审计全覆盖。进一步完善财政资金支出、土地出让、行政审批、工程项目招投标、公共资源交易等领域的监督制度。严肃问责，抓早抓小，抓日常抓经常，动辄则咎。扎实开展廉洁教育和廉洁文化建设，认真贯彻中央八项规定和省市若干意见，始终保持清廉本色。

各位代表！信阳发展的接力棒交到了我们手中，我们责无旁贷。让我们更加紧密地团结在以习近平同志为核心的党中央周围，在省委、省政府和市委的坚强领导下，求真务实，锐意进取，撸起袖子加油干，在造福信阳、添彩中原，决胜全面小康新征程中迈出更加坚实的步伐，以优异的成绩迎接党的十九大胜利召开!
